# Stakeholder perspectives on the preferred service ecosystem for senior citizens living at home: a qualitative interview study

**DOI:** 10.1186/s12877-023-04303-4

**Published:** 2023-09-19

**Authors:** Christophe Eward Kattouw, Karina Aase, Petter Viksveen

**Affiliations:** Department of Quality and Health Technology, Faculty of Health Sciences, SHARE – Centre for Resilience in Healthcare, University of Stavanger, Post Box 8600, Forus, Stavanger, 4036 Norway

**Keywords:** Service Ecosystem, Senior Citizens, Stakeholder involvement, Community Health Services, Health Services for the aged, Idealized Design Approach

## Abstract

**Background:**

Most senior citizens want to live independently at home as long as possible. The World Health Organization recommends an age-friendly community approach by transforming the service ecosystem for senior citizens and basing it on the question “What matters to you?”. However, there is limited research-based knowledge to determine the characteristics of the preferred service ecosystem from the perspectives of multiple stakeholders. Therefore, the aim of the study was to gain a deeper understanding of multiple stakeholder perspectives on the preferred service ecosystem for senior citizens living at home.

**Methods:**

Four stakeholder groups (n = 57) from a Norwegian municipality participated in an interview study in 2019 and 2020: senior citizens, carers, healthcare professionals, and managers. Data were analysed according to qualitative content analysis.

**Results:**

Overall, there was considerable correspondence between the four stakeholder groups’ perspectives on the preferred service ecosystem for senior citizens. Six themes were developed: (1) “self-reliance – living independently at home as long as possible”; (2) “remaining active and social within the community”; (3) “support for living at home as long as possible”; (4) “accessible information and services”; (5) “continuity of services”; and (6) “compassionate and competent healthcare professionals”.

**Conclusions:**

In order to adapt and meet changing needs, the preferred service ecosystem should support senior citizens’ autonomy through interpersonal relationships and involvement. Healthcare managers and decision makers should consider a broader range of practical and social support services. Municipalities should plan for and develop age-friendly infrastructures, while healthcare professionals should rely on their compassion and competence to meet senior citizens’ needs.

**Supplementary Information:**

The online version contains supplementary material available at 10.1186/s12877-023-04303-4.

## Background

Senior citizens value independence and prefer to continue living at home as long as possible [1–5]. This is in line with governmental policies [6, 7]. However, ageing may involve increasing health challenges like impaired mobility and reduced cognitive function, which may limit the ability to continue living at home. Support from family and healthcare professionals may be needed [8–10], but the carer-role can be demanding for informal caregivers [11–14] and healthcare professionals [15–18]. Furthermore, care services may pose a threat to senior citizens’ independence and dignity, in particular when they are fragmented rather than integrated [19–23]. Service providers’ focus on care tasks seems to defy senior citizens’ needs, perspectives and well-being [23–25]. Consequently, transformation of the service ecosystem for senior citizens living at home should be based on what matters to them [26–30].

A service ecosystem can be defined as “a relatively self-contained, self-adjusting system of resource-integrating actors connected by shared institutional arrangements and mutual value creation through service exchange” [31]. It recognises that value is co-created by multiple stakeholders, who may have different perspectives on institutional arrangements like rules, meanings and norms [32–36]. In this context, value and benefits are based on senior citizens’ expectations and experiences of the service delivery process rather than outcomes alone [30, 37, 38]. This aligns with the global “what matters to you” movement and should form the basis of the service ecosystem development [24, 25, 27, 39]. Additionally, health promotion and active ageing in age-friendly environments should be encouraged [29, 40–43]. Furthermore, the degree of correspondence between multiple stakeholders’ perspectives, has considerable implications for the transformation of the service ecosystem [44–46].

Stakeholder perspectives have been included in studies on integrated care for older people with frailty [22], healthcare service development within institutional care [47]; improvement of medication administration and cancer care in hospitals [48, 49]; and waiting times for breast cancer [50]. However, empirical research including different stakeholder perspectives seems scarce [30, 51, 52], particularly for the preferred service ecosystem for senior citizens living at home. In order to develop the preferred service ecosystem, further insight is needed with regards to the needs of senior citizens [53]. The aim of this study was to gain a deeper understanding of multiple stakeholder perspectives on how the preferred service ecosystem for senior citizens living at home can be described. The research question was: How can the preferred service ecosystem be described from the perspectives of senior citizens themselves, their carers, healthcare professionals and managers? This study is a part of a research project aiming to develop design proposals for the preferred service ecosystem for senior citizens living at home.

## Methods

### Study design

A qualitative design [54] was applied, with semi-structured individual interviews and focus group interviews [55, 56] including four stakeholder groups’ perspectives on the preferred service ecosystem. Stakeholders were senior citizens, carers, healthcare professionals and managers. Some of the senior citizens were also representatives of senior citizen organisations. Focus group interviews were carried out with senior citizens and healthcare professionals. Focus groups were used to facilitate interaction among participants and gain knowledge that might not have been revealed through individual interviews [55, 57, 58]. Individual interviews were carried out with some of the senior citizens who were unable to attend focus group interviews for health reasons. Managers were interviewed individually, primarily in order to avoid any power differentials arising from including them in focus group interviews with healthcare professionals. Both individual and focus group interviews aimed to gain an in-depth understanding of the participants’ perspectives [58, 59]. The consolidated criteria for reporting qualitative research (COREQ) checklist was used to report this study [55].

### Setting, sample and recruitment

This study was conducted in one of Norway’s 20 largest municipalities (> 75,000 inhabitants), including urban and rural areas. In 2019, home care services included nine community nursing teams, a reablement service team and seven senior citizen centres. Each team was led by a manager. Care was provided by nurses, social workers, skilled healthcare workers and untrained assistants. Reablement services were provided by physiotherapists, occupational therapists, nurses and skilled health workers.

Representatives from three user organisations and managers were consulted in the planning phase, in order to strengthen the study’s relevance, to consider its feasibility, and to discuss how participants could be recruited. Both representatives and managers influenced sample choice, recruitment procedures and interview questions (Table 1). For example, representatives suggested that carers should also be involved as participants in the study, as their perspectives are relevant and generally underexposed. They agreed it would be helpful to also include senior citizens without personal homecare experience to involve someone “thinking out of the box” (Table 1).

Inclusion criteria were: (a) senior citizens aged 67 or older, with current or past experiences with the homecare services; (b) senior citizens aged 67 or older without personal experiences with the home care services; (c) carers of senior citizens (e.g. spouse/partner); (d) healthcare professionals currently working within the homecare services; (e) managers of the homecare services.

**Table 1:** Stakeholder involvement in the planning of the research

Stakeholder group	Impact
Representatives from three senior citizen organisations (n = 14)	• Changes and additions to interview guide, including adjustment of interview questions • New interview groups: carers, user organisation representatives
Managers (n = 11)	• Recruit participants from the municipal resource allocation office

Managers and the municipal dementia coordinator facilitated access to recruiting senior citizens, carers and professionals (sample description in Table 2).

**Table 2:** Interview and participant characteristics

Stakeholder group type / interview type^1^.	Interview participants (n = 82)^2^	Participant characteristics
**Senior citizens** (n = 21)^3^	**34**	Age 71–90 (M 87), Female n = 16, male n = 5
Focus group	11	Focus groups: 50% received community nursing, Individual interviews: all received community nursing daily
Focus group	7	
Focus group	6	
Focus group	5	
Individual	5	
**Senior citizen representatives** (n = 5)^3^	**5**	Age 71–77 (M 73), Female n = 2, male n = 3
Focus group	5	No personal community nursing experience
**Carers** (n = 4)^3^	**5**	Age 74–80 (M 77), Female n = 2, male n = 2
Focus group	4	All spouses received community nursing
Individual	1	
**Healthcare professionals** (n = 22)^3^	**28**	Female n = 21, male n = 1
Focus group	8	Professional background: nurses n = 10, skilled health worker n = 8, physiotherapist n = 2, occupational therapist n = 1, social worker n = 1
Focus group	6	
Focus group	10	
Focus group	4	
**Managers** (n = 5)^3^	**10**	Female n = 5
Individual	10	Nurses n = 4, physiotherapist n = 1

(1) Individual interviews n = 16, focus group interviews n = 10. (2) 29 participants were interviewed more than once. (3) Number of unique participants for each stakeholder group, i.e. those who were interviewed more than once were only counted once

### Data collection procedures

Data was collected through 16 individual and 10 focus group interviews with 30 senior citizens and carers, and 27 healthcare professionals and managers. The number of participants was selected to ensure sufficient information power to answer the research question [60]. In this context, the study’s aim was broad and the participants’ perspectives could vary (“sample specificity”). Hence, a larger number of participants was considered to be necessary. Focus group interviews took place in meeting rooms in three senior citizen centres. Individual interviews were carried out at home (senior citizens and carer) or in their office (managers). As part of the research project, most participants were interviewed twice in the period from December 2019 to July 2020. Emphasis in the first interview was on the preferred service ecosystem, and the existing home care services in the second interview. This aligns with a “idealized design” approach where the initial focus is on the ideal state of the service ecosystem, prior to focusing on potential challenges [53, 61–63]. Data from both interviews was used, as participants also shared perspectives on the preferred service ecosystem in the second interview. None of the participants refused to participate. Eight professionals did not attend the second interview due to misunderstandings or sick leave. The second focus group interview with carers was cancelled due to Covid-19 restrictions. Instead, an individual interview took place in one carer’s home. Interview guides were used depending on the interview type (individual or focus group), interview stage (I or II), and stakeholder group (senior citizens, carers, professionals or managers). Key questions for senior citizens focused on their personal goals and their preferred service ecosystem. Carers, professionals and managers were also asked what matters to senior citizens (question A) as a starting point to encourage them to think about senior citizens’ needs and thereby prepare them to consider broader perspectives on the preferred service ecosystem [53] (interview guides in Table 3).

**Table 3:** Semi-structured interview guide (main questions)^1^

	Interview guide for senior citizens, including senior citizen representatives (individual and focus group interview)	Interview guide for carers (focus group interview)	Interview guide for healthcare professionals and managers (individual and focus group interview)	
Introductory remark^1^	We all have different personal goals that we strive to realize in our daily lives or attain in the future. The goals may be related to any life domain, such as hobbies, daily life, health, family or friends.	We all have different personal goals that we strive to realize in our daily lives or attain in the future. The goals may be related to any life domain, such as hobbies, daily life, health, family or friends.	We all have different personal goals that we strive to realize in our daily lives or attain in the future. The goals may be related to any life domain, such as hobbies, daily life, health, family or friends.	
Question A^1^	Think about the goals you have at the moment. The goals can be big or small, the main thing is that they are important to you. Can you please tell us what matters to you?	Think about the goals the person you are carers for might have. The goals can be big or small, the main thing is that they are important to him/her. What do you think matters to him/her?	Think about the goals citizens 67 + might have. The goals can be big or small, the main thing is that they are important to them. What do you think matters to citizens 67+?	
Question B^2^	One of the goals might be that you want to contribute with your resources or life-experience. What do you think that you can or want to contribute with?			
Question C^2^	Another goal can be that you want to live at home as long as possible. What do you contribute with in order to manage yourself at home?			
Question D	Can you please tell us what the features are of the ideal home care services?	Can you please tell us what the features are of the ideal home care services for the person you are carers for?	Can you please tell us what the features are of the ideal home care services for citizens 67+?	
Question E	What should the municipality’s home care service do in order for you to live the life you prefer?	What should the municipality’s home care service do in order for him/her to live the life he/she prefers?	What should the municipality’s home care service do in order for citizens 67 + to live the lives they prefer?	

(1) Based on Saajanaho et al. [4 page 197]. (2) Suggested by senior citizen representatives

All participants were provided written information about the project beforehand. During interviews, they were first given individual questions on a sheet of paper, to give an opportunity to reflect and make notes prior to responding verbally. Focus group participants were then asked to discuss questions in pairs, so everyone could voice their opinion and to support their reflective processes [64]. Several participants considered self-reflection and pair discussions helpful as it gave them time to reflect. Focus group interviews were moderated by the first author (CEK) in collaboration with the co-researcher (PV, KA), who also made notes. All interviews were audio recorded (64–152 min, median 85) and transcribed verbatim. Interviews were transcribed by the first author (n = 10) and a professional service (n = 16). All were checked by the first author.

### Data Analysis

Qualitative content analysis was carried out through six-stages [65]. Interview transcripts were read several times by the first author, and 11 were assessed by co-authors. The purpose was to get an initial understanding for each stakeholder group (stage 1). Preliminary insights were discussed by the authors. All three authors have extensive experience from clinical practice and/or interview studies.

All participant quotes contributing to answer the study’s aim and research question were extracted and transferred to Excel. Transcripts on senior citizens’ personal goals were extracted from interviews with senior citizens. In line with the research question, transcripts on the preferred service ecosystem were extracted from interviews with all stakeholder groups. Co-authors checked a random selection of quotes to assess data extraction quality. Transcribed texts were shortened and divided into meaning units (stage 2), condensed (stage 3) and coded (stage 4). Codes were categorized into various category levels (stage 5). Stage 2, 3 and 4 were carried out by the first author, discussed and agreed with the co-authors. Stage 5 was carried out collaboratively through several meetings, leading to six themes (stage 6). Table 4 exemplifies the analysis process.

**Table 4:** Example of the analysis process

Transcript	Condensed meaning unit	Code	Sub-category	Category	Theme
“It is very important that it is a conversation, when you need a home care service that it is really a mapping of, what are your needs.”	the importance of mapping your needs	mapping needs	mapping of senior citizens’ needs	Involvement	Support for living at home as long as possible
“That you have that dialogue. Because … I often think the municipality chooses to generalize and sort of … that’s what we offer of services.”	important with a dialogue [around your needs]	needs-dialogue	dialogue	Involvement	
“And then it may not be so adapted to me and you. And that is … I do not think it will be any more expensive if you in a way … have the necessary knowledge about the individual’s … we are different.”	not more expensive with necessary knowledge [about needs]	insight into individual needs	mapping of senior citizens’ needs	Involvement	
	necessary knowledge about the individual	knowledge about the individual	mapping of senior citizens’ needs	Involvement	
	we are different [individuals]	we are different (individuals)	mapping of senior citizens’ needs	Involvement	

## Results

Six themes were developed to describe the preferred ecosystem from the perspectives of senior citizens themselves, their carers, healthcare professionals and managers: (1) self-reliance – living independently at home as long as possible; (2) remaining active and social within the community; (3) support for living at home as long as possible; (4) accessible information and services; (5) continuity of services; and (6) compassionate and skilful healthcare professionals (Table 5). While a limited selection of participant quotes will be provided throughout the text, additional quotes may be found in additional file 1.

**Table 5:** Multiple stakeholder perspectives on the preferred service ecosystem

Themes	Categories	Description
Self-reliance - living independently at home as long as possible	NA	Senior citizens want to fend for themselves, have freedom to do what they want, and remain resourceful. Both adjustments and assistive devices in the home, and social networks (family, neighbours) may support independence.
Remaining active and social within the community	NA	Remaining physically active and involved in social activities can protect against isolation and loneliness for senior citizens. They need to have something to look forward to and be able to enjoy daily life.
Support for living at home as long as possible	Reablement	Supporting independence by uncovering needs and resources, in order to facilitate intrinsic motivation for training. Senior citizens should have sufficient time and support for goal setting. Potential consequences of passivity should be explained.
	Assistive devices	Assistive devices should be tailored to senior citizens’ needs, e.g. adaptive beds, threshold ramps, electronic medication devices, alarm systems to call for urgent help, GPS for those living with dementia. They should be swiftly delivered when needed and senior citizens should be informed about and receive training to use them.
	Practical and social support	Practical support involved carrying out daily activities such as getting in and out of bed, showering, house cleaning, and food and medicine delivery. To reduce family burden, professionals could assist senior citizens in their house for longer periods of time. Caretaker services and voluntary services are important support structures. Social support prevents loneliness and despair.
	Involvement	Involving senior citizens and map their needs to inform the organisation of the service ecosystem, including choices regarding assistive devices, to ensure the relevance of services in meeting these needs.
Accessible information and services	Information about services and access to professionals	Senior citizens should have easy access to information about available services, their rights, and assistive devices. In case of special needs senior citizens should know who to contact and accessible professionals should give unambiguous answers.
	Senior citizen centres	Senior citizen centres should have flexible opening hours depending on senior citizens’ and carers’ needs. Services should be free of charge.
Continuity of services	Timeliness and predictability	Senior citizens’ needs, biological rhythm and preferred (social) life should determine the professionals’ visiting times. Senior citizens should have the possibility to digitally book home care visits, deliver their eventual week plans, and have information about the time of arrival, and be informed in case of a delay.
	A limited number of professionals	A limited number of professionals working in smaller districts safeguards continuity of care and facilitates both interpersonal collaboration and relationship building with senior citizens and their carers. This may also prevent senior citizens from repeatedly presenting themselves and explaining needs, routines and preferences.
Compassionate and competent health care professionals	Professionals’ compassion	Professionals who visit senior citizens in their home should care about them, and have a comforting presence by being friendly, relaxed and accessible. They should offer, but not take over tasks or decide for senior citizens. They should signal to have enough time, genuinely listen, see the whole person, and be dignity-focused in the way they act and speak.
	Professionals’ competence	Professionals should promote health and trust by up-to-date competences, on medical and health-related questions, knowing senior citizens’ needs and preferences, and changes in needs. They should also be reflective and self-critical and have specific reablement competences which include a resource focus, motivating communication and support in dialogues while gently requiring efforts.

## Self-reliance – living independently at home as long as possible

Senior citizens valued their health and self-reliance. While their understanding of health was not further described, self-reliance implied living independently at home as long as possible. This was to fend for oneself, to have freedom to do what they wanted to, and to serve as a resource for others. They valued social networks which, together with assistive measures, supported autonomy. Living at home as long as possible was seen as “a matter of course”. Senior citizens seemed satisfied with their living conditions, but were willing to move to a sheltered accommodation, provided they could bring personal belongings. Being bedridden or needing better access to facilities like grocery stores and public transport were reasons to move. Senior citizens wanted to maintain freedom to move around in their home and fend for themselves, despite health challenges, or to manage with as little support as possible. This could include getting out of bed, taking a bath, or cleaning the house. They took responsibility for their health and physical exercise. They were decisive and strong-willed, not postponing but completing tasks, sometimes in spite of pain: “That I stay in shape and that I get out, that I go for a walk even if it hurts a little”. (Senior citizen 24)

Living independently was so important to senior citizens that they would rather do gardening or vacuum cleaning whilst sitting in their wheelchair, than moving to a nursing home. They sought information about exercise to support their ability to function and maintain independence. Staying updated on the latest news was important to them. To fend for themselves, they were willing to tolerate clutter, contrary to former preferences for tidiness. While some senior citizens mentioned loss of resourcefulness, others emphasized the importance of remaining resourceful. This included volunteering for kindergarten duties, homework support, or visiting lonely people. They highly valued social networks, including spouses, children, grandchildren and neighbours. Some felt dependent on their family, without whom they would need to move to a nursing home: “So if I should suddenly be left without my wife, then there are many things I will not manage. I know that if she suddenly becomes ill or harms herself in some way, then I will have to go to a [nursing]home.” (Senior citizen 25). However, senior citizens did not want to be perceived as a burden.

### Remaining active and social within the community

Senior citizens expressed a strong need for physical and social activities, to have something to look forward to, including cultural entertainment, and enjoying daily life. They considered it meaningful and a preventive measure against isolation and loneliness: “It means being together with other people. Be with other people for a few hours. It’s only once a week. Yes, it helps. It’s better to be here than to sit at home in a chair” (Senior citizen 5). Activities ranged from knitting, gardening, or listening to the radio, to going for a hike, cycling, visiting the theatre, or discussing daily life. Several activities took place within senior citizen centres, or with their children, grandchildren or neighbours.

### Support for living at home as long as possible

To continue living independently at home may imply a need for reablement. Assistive devices and an age friendly infrastructure can be used to support independence. For senior citizens who may not sufficiently benefit from reablement services and/or assistive devices, additional practical and social support may be required. Involvement and mapping of needs should ensure relevance of services in meeting needs.

Managers emphasised the importance of reablement, aiming for autonomy, self-reliance and self-control over daily life activities. Consequently, to continue living at home required from senior citizens to train and strain. Professionals should uncover senior citizens’ resources and needs and encourage them to make efforts to achieve their goals, rather than being «served on a silver platter». This also applied to psychosocial needs, e.g. anxiety and depression, camouflaged by asking for practical support. Uncovering personal goals could facilitate intrinsic motivation for training. However, some senior citizens lacked motivation if they did not set goals themselves, or if they had low expectations of their potential to improve. Instead of suggesting that senior citizens should “go easy on themselves” and let others do tasks for them, professionals should encourage discussions with them and explain potential consequences of remaining passive and the benefits of active involvement in reablement:

“To see the resources of the individual, that those who can, should have the opportunity to become as independent as possible. What resources does he or she have […] employees should ask them what is important to them […] But if you ask them, yes if you had that strength, or had a better balance, how would your everyday life have been then? And they [would then] manage to set goals more easily, [go] to the store [or] visit their daughter who lives a little further away but who has stairs for example. So then it is easier for them to reach a goal. […] We must […] explain the consequences of not doing anything, getting everyone else to do something for them. And then we also have to explain to them that it is possible to do something about it. And then the question arises, what do you think that if you continue to do as you do today, to sit in that chair, how are you then in half a year? […] get them started on the thought process.” (Manager 1).

Managers pointed out that provision of practical and social support should be planned and presented to senior citizens, initially as temporary with a goal to strengthen their independence. Otherwise, it could pose a threat to their freedom.

Professionals should aid senior citizens to set goals. Managers suggested that professionals should not disclose their titles (e.g. physiotherapist), as this might result in some senior citizens adapting responses to what they believe was expected. Sufficient time to reflect should be given and questions should be repeated regularly as their needs change over time. Goals could for example change during reablement, according to changes in capacity and self-insight, or contextual changes such as moving back home following a nursing home stay. However, in some cases acceptance of limitations in capacity was required, with accompanying service adjustments to meet senior citizens’ needs.

Senior citizens highlighted age friendly infrastructure, including suitable meeting places and transport, to support them to live at home. It could for example help them to visit family or attend GP appointments. Social meeting places could facilitate activities and prevent loneliness. Assistive devices could support senior citizens to continue living at home, by facilitating ability to function and safety. Examples included alarm systems and adaptive beds. Relevant information and training was required to enable senior citizens to use adaptive devices: “The first thing I think about is that I have to get proper training [to be] able to use it [the 113 app] properly.“ (Senior citizen 9).

Senior citizens and carers preferred personal contact with professionals over “cold robotic solutions”. Practical and social support included practical help, e.g. for personal hygiene and household activities, changing a light bulb, financial guidance, and accompanied healthcare visits. Social support was important to prevent loneliness and could include pet visitation or home visits by volunteers: “But a lot of people I’ve talked to, what they miss, is that someone sits down and eats with them. Not just sitting there with a cup of coffee and keeping them company, but that they eat.” (Carer 3).

Several participants emphasized the importance of what matters to senior citizens. However, managers explicitly pointed out that “what matters to you” should be the permeating philosophy of the preferred service ecosystem. Senior citizens’ needs should be met holistically based on what matters to them, including psychosocial needs. Contrary to a “one-size-fits-all” approach, services should be flexible and take into account different life situations and rapid changes due to health. Adapting to senior citizens’ needs and preferences required dialogue with each individual service recipient in order to accommodate aspects like their health status, and biological rhythm (e.g. “early birds” or “night owls”). According to senior citizens, municipal decision makers should seek senior citizens’ perspectives to ensure relevance of services to meet their needs. Their viewpoints should be mapped and they should be involved in development of the preferred service ecosystem. Mapping processes could take place through home visits or by involving groups of senior citizens:

“that you have a kind of user council, which has that setting in mind, with continuous improvement. I actually think that is an important message back to the municipalities. They have to make sure that there is a continuity, and that they look critically at the services they offer. That the user in a way is satisfied. […] universal design […] Is it friendly for an aging population? Is it easy to get parked? Is it easy to get to public transport? […] to build an age-friendly society.” (Senior citizen representative 4).

### Accessible information and services

Service information should be available and senior citizen centres should have flexible opening hours. Senior citizens should be informed about municipal and voluntary services, and assistive devices. This includes information about citizens’ rights, and available contact persons. According to managers, providing specific information about care decisions could facilitate senior citizens’ ability to appeal decisions they do not agree with. All stakeholders emphasized that up-to-date information should be available on municipal websites and provided by professionals upon request: “For most people who contact the municipality it is [a question of reaching] the right persons to receive information.“ (Senior citizen 25). Managers highlighted that cross-sectional collaboration between different municipal services would contribute to uniformly formulated information. During short-term hospital stays, information should be provided to senior citizens prior to a discharge to the municipality where they receive home care services.

Facilitated access to services implied adaptation of services to meet senior citizens’ needs. According to all stakeholders it should be easy to reach professionals when needed, such as homecare services or 24/7 helpline support. Managers specified that senior citizen centres should have flexible opening hours, including evening access, as this could support needs and reduce carer burden:

“To extend opening hours at the senior citizen centre. One thing is that it helps the senior citizen, but this is also to relief for relatives […] Should it be [open] a bit in the evening […] so carers [could also] engage in their own social activities.” (Manager 4).

Both professionals and managers emphasised that processing time required for assessing service requests should be limited to a minimum. For example, requests for wheelchairs should be processed immediately and senior citizens moving to a nursing home should not be placed on a waiting list.

### Continuity of services

Timeliness and predictability, and a limited number of professionals constitute the elements of continuity of services for senior citizens described in the current study. Services provided at agreed times by a limited number of professionals for each individual senior citizen, would contribute to support security and trust through predictable services, and strengthen interpersonal relationships and collaboration between senior citizens and professionals. This was particularly important for senior citizens with specific care needs (e.g. with dementia).

Continuity would limit the number of different professionals who senior citizens would communicate and become familiar with. It would reduce professionals’ need to travel, as different care tasks for a senior citizen could be carried out by one professional during the visit. Several stakeholders said it was difficult to specify the ideal number of professionals caring for a senior citizen. Some suggested between one and fifteen professionals. Limiting the number of professionals for each senior citizen would contribute to improved service continuity:

“I want one person to come, not too many, preferably the same. You may need help to bathe, put on clothes, and things like that. That there is no different person every time. And to feel that you have trust. I would say max 2.” (Senior citizen representative 2).

Continuity could be achieved through smaller healthcare districts for smaller populations, and a larger proportion of professionals working fulltime, as opposed to more professionals working parttime. Easy access to “primary contacts” with responsibility for the entire care path would provide predictability and support continuity in follow-up. Sufficient time should facilitate professionals in doing a proper, safe and sound job. Professionals would get more time to sit down and give senior citizens sufficient time to express feelings or ask questions.

Senior citizens should have the possibility to digitally book visits and provide their week plans to home care services. This would require adaptation of professionals’ rotation schedule to better meet senior citizens’ needs. For some senior citizens, delay of the service delivery of 30–60 min was acceptable, although prior notification should be given. However, in the event of sudden and urgent needs, services would be required instantly: “come when he has to go to the toilet. We always manage to hold, maybe for a quarter of an hour, maybe twenty minutes, but we do not manage a whole hour. That is not dignified” (Carer 2).

### Compassionate and competent healthcare professionals

Professionals’ compassion and competence were essential elements of the preferred services. Senior citizens, carers and professionals emphasised the importance of compassion, managers highlighted reablement competence.

Compassionate professionals should be caring, comforting, service minded, relaxed and respectful. They should genuinely care about their job and about senior citizens. They should be friendly and clearly indicate they have time for the senior citizen, also to answer possible questions. This implied sitting down and a relaxed behaviour, as opposed to appearing stressed and being in a hurry. Dignity should be safeguarded by respectfully talking to senior citizens as “equals”, being sensitive to their needs, and checking how they experienced the services. They should involve senior citizens and respect their preferences, and strive to get to know them. This involved treating them as whole persons, rather than patients with diagnoses:“ […] see the whole person. They should treat them as a person, not as a diagnosis.” (Skilled health worker 22).

To do so, they should find out what matters to them and show genuine interest in their personal stories. They should give senior citizens time and space to respond to questions, and put forward their requests. They should also respect the difference between a private home and an institution. This involves e.g. taking off shoes or using shoe covers, and not use their phone for private calls during visits. Professionals should be cleanly and language skills were important, especially if senior citizens might have acute health problems.

Professionals should be competent to notice development and changes in senior citizens’ needs through close monitoring and continuous dialogue. They should also reflect and possess a critical sense in order to continuously assess if and how they should execute certain tasks. Trust was also strengthened when professionals obtained updated information about senior citizens; they demonstrated professional skills; were time efficient; and were in control of extraordinary situations, for example when severely sick senior citizens were discharged from hospital. According to managers, reablement competences involved professionals who were reflective, flexible, creative and persistent in finding solutions, which could also include the use of assistive devices. Through their dialogue and communication, they should motivate senior citizens to reablement and training in line with their personal goals. Professionals should also be able to assess and monitor senior citizens’ function as well as progress during training, to make the necessary adjustments:

“That they stop and react or they wonder why is it written that we should go in and do this and that, when he can manage himself […] They should learn to see the resources of the individual and that those who can should have the opportunity to become as independent as possible […] When you train to get better at something then you need to know how the status was yesterday. If you have not been there for a week, then you cannot know that. You can then not take action to make [the training] more difficult or challenging or see whether there was a change […].” (Manager 1).

## Discussion

This study is unique in reporting the perspective of four different stakeholder groups (senior citizens, carers, healthcare professionals, and managers) on the preferred service ecosystem for senior citizens living at home. In order to describe this service ecosystem, six themes were developed: (1) “self-reliance – living independently at home as long as possible”; (2) “remaining active and social within the community”; (3) “support for living at home as long as possible”; (4) “accessible information and services”; (5) “continuity of services”; and (6) “compassionate and competent healthcare professionals”.

First and foremost, we found considerable overall agreement on the preferred service ecosystem among the four stakeholder groups, while discrepancies existed regarding the focus on an age-friendly infrastructure and reablement. The service ecosystem should facilitate senior citizens to live their desired lives, which involves living independently at home as long as possible and being active and social within the community. An age-friendly infrastructure implies suitable meeting places and transport, and autonomy can also be supported by the use of assistive devices. Temporary healthcare services in the form of reablement are preferable, as they aim for independence. For senior citizens needing long-term practical and social support, clear information about available services should be provided. These services should be easily accessible and timely, characterised by continuity, and executed by compassionate and competent professionals. The preferred service ecosystem should be adaptive and continuously involve senior citizens in its development and service execution.

The agreement among the four stakeholder groups in this study can be perceived as a good starting point for the development of a preferred service ecosystem for senior citizens, indicating a consistency in assumptions and values [36, 66]. A possible explanation for the similarity between the different stakeholder groups may be that healthcare professionals work closely with senior citizens over extended periods of time, and managers also have past experience from the practice field, within the context of senior citizens’ homes. Although health and home care systems in other countries, such as the Alaskan Nuka system [67] and the Dutch “Buurtzorg” home care model, display some of the characteristics of an adaptive service ecosystem [27, 67–72], our findings emphasize additional important elements, such as age-friendly infrastructures and reablement.

Senior citizens’ need for self-reliance and remaining active and social as long as possible has also been identified in other studies, e.g. confirming how senior citizens felt responsible for making adjustments in their home, managing themselves despite pain and/or health challenges, and the dependence on others [6, 8]. Our study adds that senior citizens emphasised that despite their support needs, they do not want to be perceived as a burden.

Senior citizens particularly highlighted age-friendly infrastructures, including suitable meeting places and transport. This aligns with two of the WHO’s eight age-friendliness domains [29]. However, other age-friendliness domains like social participation, inclusion, respect and a positive view of ageing should also be part of the preferred ecosystem for senior citizens [29, 73, 74]. This also corresponds with our findings, where senior citizens expressed the need for being perceived as a resource, and the desire to be involved in the development of the preferred service ecosystem. An explanation for why only senior citizens highlighted age-friendliness domains like infrastructure and transport might be due to differences in stakeholders’ perspectives. Previous research suggests that senior citizens focus more on their strengths and health promotion, while healthcare professionals rather emphasize disease and risk prevention, underestimating senior citizens’ qualities and aspirations [75, 76].

Support for living at home should be provided through reablement, assistive devices, and practical and social support. Senior citizens should be involved in the development and implementation of the preferred service ecosystem. In our study, managers particularly emphasized the importance of reablement as it aims to accommodate senior citizens’ need to live independently at home as long as possible. Managers are more likely to be better informed about the municipal policies. They may also regularly discuss reablement as an important measure to strengthen senior citizens’ autonomy and reduce the need for practical and social support. Managers suggested that healthcare professionals might focus less on reablement, due to time pressures in delivery of supportive care. Why the senior citizens did not mention reablement may be because the concept appears abstract and goal setting can be challenging [77–79]. In line with previous research, managers in our study also suggested that healthcare professionals should have in-depth understanding of reablement and be able to clearly explain it to senior citizens [80].

The senior citizens’ suggestion to eventually use assistive devices or to change housing to an area with appropriate infrastructure aligns with findings on ageing in place [8]. Furthermore, our and previous research suggest that information and communication (technology) are important within the context of the preferred ecosystem [74, 81, 82]. However, though assistive devices might support independence, the senior citizens in our study expressed ambivalence towards adopting technologies and sensors. Other studies have confirmed this, referring to lack of trust, limited experience, higher age or cognitive impairments as reasons [83, 84]. In line with the suggestions of the senior citizens in our study, they should be closely involved in both the choices and the implementation of assistive devices and technology.

Previous research highlights the absence of a clear definition of continuity of services. To address this gap, it has been suggested that the continuity of services involves provision of coordinated care and services that are consistent with patients’ health needs and personal circumstances, delivered over time and across different levels and disciplines [85]. However, this definition primarily focuses on the existing healthcare services and does not consider the preferred service ecosystem. Moreover, it does not address important aspects such as timeliness and predictability, which our study contributes to.

The concept of “the preferred service ecosystem” has not been applied in previous studies. Moreover, although topics such as autonomy, mutual trust and relationships, professionals’ compassion and competences, and accessible information have been identified in other studies [1, 2, 8, 22, 86–98], the majority of these studies describe the perspectives from only one or two stakeholder groups (mostly senior citizens and/or carers), often excluding the perspectives of healthcare professionals and managers.

### Methodological considerations

We involved four stakeholder groups and supported our findings with detailed descriptions. Although vulnerable senior citizens (higher age and more comorbidities) were included in the study, a larger proportion of these stakeholders could have provided additional findings to better meet these citizens’ needs. Though supported by senior citizens representatives, the “what matters to you” question used in the interviews was perceived as abstract and difficult to answer for some senior citizens. As a consequence, some latent needs may have been missed. Possibly, visual and tangible communication tools (e.g. pictures), could have been used to better gather their perspectives. Although transferability of the results could be considered limited due to data collected from a single Norwegian municipality, the results correspond with previous published studies internationally. Most of the previous studies did however only report results from the perspectives of senior citizens, and not from the perspectives of multiple stakeholder groups.

## Conclusions and implications

The four stakeholder groups in this study were consistent in their understanding of the preferred service ecosystem for senior citizens living at home, while discrepancies existed regarding the focus on an age-friendly infrastructure and reablement. The service ecosystem should enable senior citizens to live independently at home as long as possible and being active and social within the community. In sum, the service ecosystem should be adaptive and continuously involve senior citizens in its development and service execution. Vital aspects include an age-friendly infrastructure, autonomy supported by assistive devices, reablement to support independence, clear information on available, accessible and timely services characterised by continuity, executed by compassionate and competent professionals.

Our study implies that multiple stakeholders should be involved in community-based service development. Healthcare managers and decision makers should consider a broader range of practical and social support services. Municipalities should plan for and develop age-friendly infrastructures, while healthcare professionals should rely on their compassion and competence to meet senior citizens’ needs. The study findings may also be useful for housing corporations and welfare organisations. In addition, the study results may serve as useful input to healthcare education, recruitment strategies, and supervision structures. Development of the preferred service ecosystem has the potential to accommodate what matters the most to senior citizens and their carers. Further research is warranted to determine how age-friendly infrastructures may contribute to this development. Moreover, healthcare professionals’ limited focus on reablement within the context of the existing homecare services should be explored.

### Electronic supplementary material

Below is the link to the electronic supplementary material.

Supplementary Material 1

## Data Availability

The datasets used and/or analysed during the current study are available from the corresponding author on reasonable request.
