# Supplementary material for: Stakeholder perspectives on the preferred service ecosystem for senior citizens living at home: a qualitative interview study
Source: BMC Geriatr. 2023 Sep 19;23:576. doi: 10.1186/s12877-023-04303-4 (PMC10508029; doi:10.1186/s12877-023-04303-4)
Supplement: Supplementary file 1 — Supplementary Material 1 [file 12877_2023_4303_MOESM1_ESM.docx]

| **Additional file 1: additional illustrative quotes on the preferred service ecosystem** | | |
| --- | --- | --- |
| **Themes** | **Categories** | **Illustrative quotes** |
| Self-reliance - living independently at home as long as possible | NA | To stay at home as long as possible. One wants to stay at home. To manage as much as possible by oneself (Senior citizen 8) |
|  |  | To do the things that has to be done. [e.g.] It is important to be stubborn and willing to persevere with what has to be done in the home for as long as possible. To do what I can do as long as possible (Senior citizen 16) |
|  |  | change to housing with everything on one level. You cannot live in a home where you have to deal with three floors […] And you have to arrange for a wheelchair. (Senior citizen representative 1) |
|  |  | I want to be a resource, instead of a burden to society. And convincing the different generations that we have something to learn from each other, and we can help each other in different ways. (Senior citizen representative 3) |
| Remaining active and social within the community | NA | Being able to do exactly what you want. […] The community is important. Just being together with people, you know. That is also very stimulating (Senior citizen 12) |
|  |  | Yes, it is social. I have been exercising, and think exercise is very important. So I've been walking a lot, and I'm involved in most activities (Senior citizen representative 2). |
|  |  | I've had a heart attack, but I'm nevertheless fine. I am very grateful that I have good health, and can walk in the garden, and work and such. (Senior citizen 4) |
| Support for living at home as long as possible | Reablement | Obviously it can change [...] when they are on a short-term stay in a nursing home and we have a network meeting, it may be important for the patient just to be allowed to come home [...] when he has arrived home, there is something else that is important, for him, since he has achieved it and then it may be to be able to start at the senior citizen centre or manage to shower all by himself, or things shift a little based on what goal achievement you have [. ..] it can probably sometimes be experienced by service users as a very big question [what matters to you?] which is sometimes a little difficult to answer right away. So it's a bit like that patients sometimes need some time to answer it and think… (Manager 3) |
|  | Assistive devices | I have such high thresholds out on the balcony, and to the bathroom. I who have stumbled so easily and break bones. Then I have mounted an inclined [threshold eliminator] from the threshold down to the floor in the hallway, so it is easier to get the wheels or the walker up, it is easier for me to get in. (Senior citizen 14) |
|  |  | Sufficient assistive devices, not just that you got granted, but that you actually got it when you needed it. (Nurse 18) |
|  | Practical and social support | Among other things [care] of feet one might be bothered with, and that they could at least cut nails. (Senior citizen 18) |
|  |  | His hand is lame, and has to be washed. It's just like feet that are not washed. If the hand is not washed, you get a bad smell in it. These are 'basic things' I'm talking about. Being able to get on the toilet, it has to be a human right. (Carer 2) |
|  |  | If I were to grow old, I would shower every day. I would not shower once a week. (Manager 5) |
|  |  | Home visits by the doctor, that it is easier to get, it should definitely be possible. And preferably psychologists, that they could come home. Too many people struggle with just getting out with poor health. (Nurse 4) |
|  |  | if they want this, that they should be allowed to do it in the shower. Then they really have to be allowed to say this […] They still have their [sexual] needs too. (Skilled health worker 20) |
|  |  | more help with finances, that is, who will teach you to pay bills online or things like that. (Manager 4) |
|  |  | Volunteers are very important in the future and we are working to get more and more offers (opportunities?) and maybe help to get in touch. Such an activity friend, and takes people out on occasions, what they want, that they get some content (substance?) in their lives. (Manager 4) |
|  | Involvement | That [the municipality / political decision makers] might have had more insight into the ordinary, the daily, how we feel at home. That maybe someone could go home sometime, and interview or something, or talk to them. (Senior citizen 18) |
|  |  |  |
|  |  | We can not be pushed bits and bobs from the municipality and suppliers that are not relevant and often do not provide good enough benefits for us. As a user, we should also have influence on what we get and what we need. (Senior citizen representative 1) |
| Accessible information and services | Information about services and access to professionals | That we get an overview of everyone who runs that service. (Senior citizen representative 3) |
|  |  | It is important that you have access to the services you may be entitled to. And that he should not have to be referred and referred, but that there should be good information on those webpages and one should meet the right person on the numbers that are there, and that things are updated then, what the municipality can offer. And it does not have to be such a disease-like thing, it can be a tour group [...] that the services were perhaps more flexible. That is, the citizen reports a need, and then that need is received, and then one finds out about it and comes with a feedback related to it. That maybe not the person has to somehow take all this contact. Less silo activities. (Manager 4) |
|  |  | They can call in, the hotline is always open, we have extremely many phones, so we are in a way, we are a 24/7 phone, SOS phone. (Manager 5) |
|  |  | That [employees] can in a way obtain information quickly, so that if there should be situations that require a quick overview, that one is confident that the nurses can get that information, if it can make the citizens best taken care of in such situations. (Manager 3) |
|  | Senior citizen centres | It makes it on an equal footing, because it costs nothing, so it's probably easier to accept help then. (Nurse 8) |
| Continuity of services | Timeliness and predictability | It is one of the most important things that they keep agreed times, whether it is a toilet visit, or if there is something else. Otherwise you get collisions with other appointments. That they [leaders] also set up a time schedule that is executable, which contains times that they can physically reach. (Senior citizen 25 |
|  |  | That they can get up whenever they want, they can go to bed whenever they want [...] And we have people who are going to the senior citizen centre who need to be ready for the bus to come, yes, people who go to the hospital and have an appointment there (Manager 5) |
|  |  | And then there are others who want to attend social events [with family/friends], and want to contribute, it is important for them that we arrange the service so that they can participate in what they want, that the services do not in a way put a damper or makes it impossible to do what you want to do in your life. The ideal must be that if I was 67 years old and needed something, that I could set up this plan myself. How I want it to be, what time I want them to come, that I have set up a weekly plan... (Manager 4) |
|  | A limited number of professionals | that there are not so many who come, one every day, different people all the way, to avoid to get to know. So it is very nice to stick to, two, three (Senior citizen 8) |
|  |  | about permanent staff, if one is very ill and needs a kind of reassurance that you know who is coming, you know them, you know that they know what they are doing. But also considering the time they expect help and that one kind of keeps that. That they kind of know what the day brings. At least the oldest and the most frail, sort of, there is so little it takes to tip them off the stick. And just like you never get any such chemistry with that person, does not get to know. (Nurse 4) |
|  |  | familiar persons, yes. So I think smaller zones due to all those things both for employees, medicine management, and to get to know, so that the users will not have so many to relate to. Then there will also be fewer then, at least had a familiar person who followed them up on that [...] that you have the primary contact from a to z. From the moment you enter [the healthcare system] until the end (Manager 2) |
| Compassionate and competent health care professionals | Professionals' compassion | That they friendly and nice and kind, that they ask can I help you with something?, That they are not upset and angry and scold you and so […] that I can say so and so without them being angry and offended at me. […] That we can discuss something, because we may start talking about something while they dress me. And then we were not done and then we continue a little bit. (Senior citizen 24) |
|  |  | That he is relaxed. I notice it very well on his hand, the cases where he has been really satisfied and has had a good time, then the hand is completely relaxed [That the professionals] talks nicely with him. Rather dicusss. So not impersonal and not in a way that they are too busy. To get to know that person as he really is. (Carer 2) |
|  |  | That you feel talked to as an equal person, not necessarily as a patient. It gives me the impression that they care about what they are doing. That they also get feedback from the patient if what they are doing is okay, ask if you are standing OK [in the Active sit-to-stand lift], if it hurts in the leg or something like that. And that you are told that you must say it as soon as you feel that something is unpleasant or painful […] That they have a fresh smell. That they have brushed their teeth. [...] when they change clothes in a way that makes you feel that they are not working too fast so that they tear and struggle a bit in it. That they treat my body and my clothes in such a way that it is done skilfully, and that they do not make you hurt afterwards […] It is called in English «tactile» […]. And then it's a bit up here [eyes] too. (Senior citizen 25) |
|  | Professionals' competence | That we could think of more health support, i.e. salutogenesis. Illness is illness, and that's okay, but we have to think people should be healthy. What makes them healthy. (Nurse 8) |
|  |  | So you have to know it, because it can not be the case that they come to me and then they ask me, what is wrong with you? We are so different as individuals. And I do not think it will be any more expensive if you in one sense have the necessary knowledge about the individual [...] but it turns out perhaps, that, the service, the need, it changes over time. And the ability to perceive that, in fact, now some changes must be made, now it there have to be some adjustments, now service need is different. And I think it will be important for the municipality, that they are able to see the need for change [...] Because I think that part of the key to getting a good service is that you have highly qualified people [...] So I believe that one must have a critical focus on having the right competences at all times when it comes to municipal services. (Senior citizen representative 4) |
|  |  | But we want to know what that ointment is good for. What do you use it for? Things like that. That you get proper answers back, it means a lot. And the tablets and stuff, especially (Senior citizen 8) |
|  |  | In general, I feel that they should have both the technical and the human part of the education before they are allowed into private homes [...] It is in a way an insight into the human mind and the human psyche. And you can have a natural ability for it. just like you are good at math. You can have a natural ability but not everyone has, but you have to let it be a part of your profession, in a profession like this (Carer 2) |
